# Anterior Auditory Field Is Needed for Sound Categorization in Fear Conditioning Task of Adult Rat

**DOI:** 10.3389/fnins.2019.01374

**Published:** 2019-12-20

**Authors:** Zhiyue Shi, Sumei Yan, Yu Ding, Chang Zhou, Shaowen Qian, Zhaoqun Wang, Chen Gong, Meng Zhang, Yanjie Zhang, Yandong Zhao, Huizhong Wen, Penghui Chen, Qiyue Deng, Tiantian Luo, Ying Xiong, Yi Zhou

**Affiliations:** ^1^Department of Neurobiology, School of Basic Medical Sciences, Army Medical University, Chongqing, China; ^2^Department of Materials Science, Chongqing University of Science and Technology, Chongqing, China

**Keywords:** auditory cortex, anterior auditory field, fear conditioning, sound recognition, chemogenetic deactivation

## Abstract

Both primary auditory cortex (A1) and anterior auditory field (AAF) are core regions of auditory cortex of many mammalians. While the function of A1 has been well documented, the role of AAF in sound related behavioral remain largely unclear. Here in adult rats, sound cued fear conditioning paradigm, surgical ablation, and chemogenetic manipulations were used to examine the role of AAF in fear related sound context recognition. Precise surgical ablation of AAF cannot block sound cued freezing behavior but the fear conditioning became non-selective to acoustic cue. Reversible inhibition of AAF using chemogenetic activation at either training or testing phase can both lead to strong yet non-selective sound cued freezing behavior. These simple yet clear results suggested that in sound cued fear conditioning, sound cue and detailed content in the cue (e.g., frequency) are processed through distinct neural circuits and AAF is a critical part in the cortex dependent pathway. In addition, AAF is needed and playing a gating role for precise recognition of sound content in fear conditioning task through inhibiting fear to harmless cues.

## Introduction

Previous studies suggested that auditory cortex can be divided into different subregions according to anatomical connections (e.g., thalamocortical projection) and functional differences (e.g., tonotopic map) (Rutkowski et al., 2003). For example, the rat auditory cortex can be divided into at least four regions: the primary auditory cortex (A1), the anterior auditory field (AAF), the posterior auditory field (PAF), and the ventral auditory field (VAF, also known as secondary auditory cortex or A2 in some literature) (Doron et al., 2002; Shiramatsu et al., 2016; Tao et al., 2017). Among these subregions, A1 and AAF in rat are largest in size [A1 = 1.35 ± 0.16 mm^2^; AAF = 1.21 ± 0.13 mm^2^ according to Polley et al. (2007)] suggest both A1 and AAF are essential for auditory perception. Anatomical evidences revealed that thalamocortical projections to AI and AAF could be originated from different divisions of medial geniculate body (MGB) (Andersen et al., 1980; Morel and Imig, 1987; Lee, 2004). A double-labeling study in A1 and AAF using retrograde tracing found that less than 2% of thalamocortical projection neurons terminate in both areas (Lee, 2004). These results suggested that although A1 and AAF are similar in hierarchy of auditory pathway (Rouiller et al., 1991) they may have distinct physiological significance for auditory processing. Compared with the extensive research conducted on A1, our current understanding about the role of AAF in auditory perception is much less clear.

Previous studies have revealed that the primary auditory cortex (A1) and AAF are both tonotopically organized in rat but with distinct patterns (Doron et al., 2002; Polley et al., 2007; Tao et al., 2017). Clear tonotopic gradients can be found in both A1 and AAF which can be used to separate A1 and AAF. Neurons with low characteristic frequency (CF) are mainly distributed in the posterior-ventral (caudal) part of Al, and neurons with high CF are mainly distributed in the anterior-dorsal (rostral) part of A1. This gradient reverses in AAF, with CF gradually decreases from caudal to rostral (Lee, 2004). Taken together with the heterogeneous thalamocortical projection in A1 and AAF, the function of AAF and A1 in auditory perception could be different yet closely linked. Previous studies in cats and rats both suggest that there are direct excitatory corticocortical projections from A1 to ipsilateral AAF (Tao et al., 2017). In cats, AAF deactivation using reversible cooling can suppress tone-evoked responses in ipsilateral A1 (Carrasco and Lomber, 2009), and auditory cortex (including A1 and AAF) deactivation can modulate responses of contralateral AAF (Carrasco et al., 2013). In rat, optogenetic activating neurons in A1 can enhance the sound-evoked responses by lowering intensity threshold and broadening bandwidth of frequency tuning in AAF, and A1 deactivation can result in opposite effects (Zhang et al., 2017). In cat auditory cortex, it was proposed that fields rostral to primary auditory cortex (comparable to AAF in rat) could be specialized for processing of auditory pattern and auditory fields caudal to primary auditory cortex (comparable to PAF in rat) could be specialized for accurately determining the spatial location of a sound source (Romanski et al., 1999; Tian et al., 2001; Boatman and Kim, 2006; Lomber and Malhotra, 2008). Yet, little is known about the role of AAF in rat in the processing of content of auditory information especially its behavioral significance.

In this study, we modified classic sound cued fear conditioning to train the rat to categorize two sound cues: a dangerous cue and a safe cue. After training, rats with intact auditory cortex can behave accordingly (freeze or not freeze) when different sound cues were delivered. Then we tested whether AAF is needed for correct categorization in this behavioral task. After bilateral ablation of AAF, the categorization ability of rat dropped significantly. Reversible silence of bilateral AAF using chemogenetic approaches (DREADD) during training or testing phase was performed to further unravel the role of AAF. Results showed that silence of AAF either during training or testing phase would significantly decrease the categorization performance of rat. Because AAF inactivation did not block the freezing behavior caused by sound cues, this suggested an AAF-independent pathway might be involved in sound evoked fear condition. And AAF plays an important role in categorization of sound frequency during this specific behavior.

## Materials and Methods

### Animal Preparation

Adult Sprague–Dawley rats (female, 2 months, 200–240 g) were provided by the Laboratory Animal Center at the Army Medical University. All experimental procedures were performed in accordance with institutional animal welfare guidelines and were approved by the Army Military Medical University Animal Care and Use Committee. In this study, animals (adult rats) were maintained on a 12-h light/dark cycle with free access to food and water at a room temperature of 25–28°C. All efforts were made to minimize animal suffering. Minimum number of animals required for statistical reliability were used. Every experimental procedure was executed in accordance with the National Institutes of Health Guide for the Care and Use of Laboratory Animals and was approved by the Army Military Medical University Ethical Committee for Animal Research (SYXK-PLA-20120031).

### Functional Localization of AAF

**FIGURE 1:**
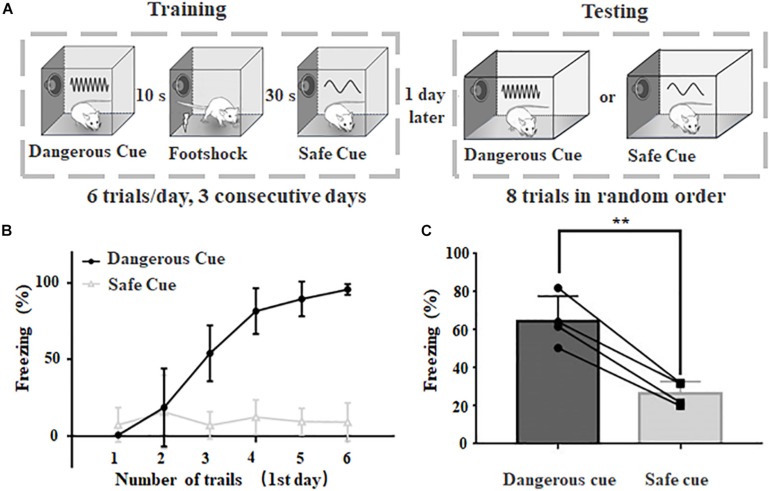
Behavioral paradigm to categorize dangerous and safe sound cues in rats. **(A)** Schematic drawing of training and testing protocol. **(B)** Freezing duration to sound cues during training session in the first day. **(C)** Freezing duration to dangerous and safe cues during testing session. ^∗∗^*p* < 0.01, paired *t*-test.

A mixture of ketamine (55 mg/kg) and xylazine (6.4 mg/kg) was used for initial anesthesia. Following anesthesia (ketamine, 13 mg/kg) was used every 30–45 min based on animal’s response to paw clip. Lidocaine (2%) was used for local analgesics. After craniotomy above auditory cortex, a tungsten electrode (0.1 MΩ, WPI Inc., United States) was used to measure the tonotopic map at a depth of 450 μm. A high precision magnetic speaker (MF1, TDT Inc., United States) was used in this study and sound pressure levels were calibrated using a 1/4” pressure prepolarized condenser microphone setup (377A01 microphone, 426B03 preamplifier with 480E09 signal conditioner, PCB Piezotronics Inc., United States). For electrophysiological recording, TDT system 3 (TDT Inc., United States) were used. This includes RP2.1 enhanced real-time processor, RA16 Medusa base station with RA4PA 4 channel preamplifier. A commercial software (Brainware, TDT Inc., United States) was used for *in vivo* recording. Tungsten electrodes with an impedance between 0.09–0.12 MΩ were used for multiunit activities (MUA) recording. A small Ag/AgCl pellet was used as reference electrode. Short pure tones (25 ms duration with 10 ms sinusoid ramp, 100 ms inter stimulation interval) consists of different frequencies and intensities (0.5–64 KHz with 0.1 octave step; 0–70 dB with 10 dB step) were used to map the characteristic frequencies in auditory cortex and locate AAF based on the tonotopic map. The tonal receptive field (TRF) was obtained for each recording site. Then, the tonotopic map was reconstructed based on the CF of each recording site. The CF increases from caudal to rostral in A1 and decreases in AAF (Tao et al., 2016). Because the high CF region of A1 and AAF are colocalized, we chose to investigate the low-mid frequency (<10 kHz) region in AAF in this study. The typical distance between the low-mid frequency region of A1 and AAF is ∼2 mm (Figure 1).

### Behavior Test

Classic sound cued fear conditioning was used in this study (Figure 1A). Two different chambers were used for training and testing, respectively. Both the training chamber and the test chamber were cleaned with 70% ethanol before and after each session. An HD camera was mounted on the ceiling of each chamber to record animal behavior. The freezing was scored when no movement (except for respiratory movements) was lasted for at least 1 s, and the total freezing time during a sound presentation was counted based on video analysis. The captured video was further analyzed by individuals who are blind to treatment.

#### Training

The rat was placed in the training chamber and allowed to explore for ∼5 min for habituation purpose. Following a 20-s continuous sound cue (“danger cue,” 70 dB 9 kHz pure tone) and a trace period of 10 s, a mild foot shock (1.0 mA direct current/DC, 2 s) was delivered. 30 s later, a 20-s continuous sound cue (“safe cue,” 70 dB 2 kHz pure tone) was delivered. Six trials were repeated, with an inter-trial-interval of 600 s. After six trials, the animal was returned to its home cage using a transfer cage. Animal was trained continuously for 3 days.

#### Test

Twenty-four hours after the third training session, a different transfer cage was used to transfer the test animal to the testing chamber with different context. The rat was allowed to explore the chamber for 3–5 min. Then, a 60-s continuous sound cue was delivered, which was the same as the dangerous cue or safe cue used in the training session. A total number of eight trials (four dangerous cue trials and four safe cue trials in random order) were repeated. The inter-trial-interval was about 10–30 min which was determined by the recovery of freezing behavior. The variable inter-trial interval during testing session (10–30 min) was due to the individual difference of each animal. Some animals can rapidly recover from the freezing status caused by warning sound presentation and others might take longer to recover. A minimal interval of 10 min was given even if there is no freezing behavior (e.g., safe sound was delivered).

Please be noted that during training sessions, animals might use different cues (e.g., sound frequency, sound sequence) to categorize different sound cues because sound cues are delivered in a fixed order. During testing sessions, we shuffled the sound sequence and changed the duration of sound presentation to make sure animal can only use sound frequency to categorize different cues.

Videos were captured using an infrared camera (Jingshiwei, China) with a frame rate of 20 fps and a resolution of 1280 by 720. For behavior response, a blind procedure was implemented for behavioral analysis. The freezing behavior was scored when no movement (except for respiratory movements) was detected for at least 1 s based on video analysis. And the freezing percentage was calculated as the ratio between total freezing time during sound presentation and the whole duration of sound presentation. The percentage is calculated based on the behavior of each animal.

### Surgical Ablation of AAF

Adult Sprague–Dawley rats were anesthetized with a mixture of ketamine (55 mg/kg) and xylazine (6.4 mg/kg), and a small craniotomy (∼1.5 mm^2^) was performed in the presumed location of AAF (“3.8 mm posterior to bregma” to “5 mm posterior to bregma,” a total distance of 1.2 mm) on the temporal skull. Then tonotopic distribution within the craniotomy window was obtained and the opened window was adjusted to locate the low-mid frequency region in AAF (1–10 kHz). Cortical tissue was then gently removed using suction pipette attached to a medical vacuum until white matter was visible. Gel foam was then used to contain bleeding and cover the ablated area. Recover the temporal muscle and suture the skin. Apply antibiotic ointment to minimize the risk of infection. After the animal is recovered from anesthesia, return it to its housing cage. For animals with AAF ablation surgery, we waited 2 weeks (14 days) for animals to recover.

### Chemogenetic Manipulation

Adult Sprague–Dawley rats were anesthetized with a mixture of ketamine (55 mg/kg) and xylazine (6.4 mg/kg), and a small craniotomy (1 mm^2^) was performed in the presumed location of AAF (3.8–5 mm posterior to bregma) on the temporal skull. Then we obtained TRFs within the craniotomy window and adjusted the craniotomy window to make sure the CF of the injection site was within the 1–10 kHz. Once we located the lower-to-middle frequency region of AAF, the following adeno-associated virus (pAAV-hSyn-HA-hM4D(Gi)-mCherry or rAAV-Ef1α-EYFP-wPRE-PA) was used depending on the purpose of the experiments.

The virus was injected using a glass pipette with broken tip (opening: ∼20 μm) attached to a micro pump (UMP3, WPI Inc., United States). For each injection, 0.5 μl of virus (titer: ∼1.2 × 10^13^ for hM4Di, ∼5.63 × 10^12^ for rAAV-Ef1α-EYFP-wPRE-PA) was injected at a rate of 25 nL/min. After each injection, the pipette was left at the injection site for 10 min before withdrawal. For craniotomy window larger than 1 mm^2^, the opening will be covered using Kwik-Cast Sealant (WPI Inc., United States). Then, the scalp was sutured, and the animals were returned to their home cages. The animals were allowed to recover for at least 4 weeks after virus injection. After recovery, extracellular physiological recordings were used to verify the effects of chemical genetic viruses. For electrophysiological response, a threshold set at three times the standard deviation above base line (50 ms window before stimulation onset) was used to detect MUA. The peak response in a 50 ms window after stimulation onset was calculated to quantify the evoked response.

### Immunohistochemistry

Immunohistochemistry reactions were used to investigate the specificity and efficiency of the expression of AAV. Four weeks after the injection of the virus, the brain was cut into frozen sections (CM1900 cryostat, Leica GmbH, Buffalo Grove, IL, United States) at a thickness of 35 μm. Sections were mounted onto glass slides, and fluorescent images were collected using a fluorescent microscope (Olympus BX53, United States).

## Results

### Rats Can Categorize Dangerous and Safe Frequency in Fear Conditioning Task

A behavioral protocol was modified from classic fear conditioning paradigm to train rats to behave selectively (freezing or not) when dangerous or safe cues were delivered (Figure 1A). The dangerous cue and safe cue were 9 and 2 kHz pure tones at 70 dB SPL, respectively. The animal was trained six trials a day in the same training chamber for three consecutive days and tested on the 4th day in a different chamber (see section “Materials and Methods” for details). Animals with normal hearing can rapidly learn to behave correctly to different cues (Figure 1B), even in the first day. After 3 days of training, animal’s freezing behavior become stable when either sound was delivered (dangerous cue: 64.6 ± 6.6%, safe cue: 26.4 ± 3.2%, *n* = 4, *p* = 0.0033, paired *t*-test, Figure 1C). The results suggested that after training, rats can categorize dangerous and safe cue in fear conditioning task.

### Bilateral Ablation of AAF Impairs Animal’s Categorization of Sound Frequency

**FIGURE 2:**
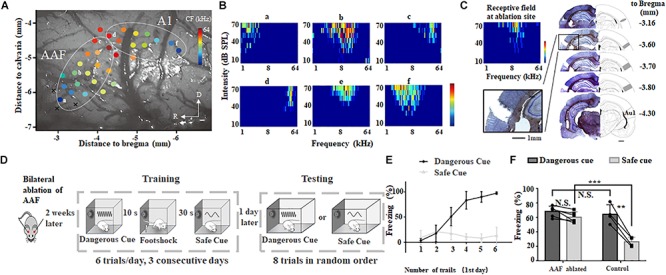
Bilateral ablation of AAF impairs animal’s categorization of dangerous and safe cue. **(A)** A representative tonotopic map in the left auditory cortex. Color dot shows the characteristic frequency (CF) at recording site. A1, primary auditory cortex; AAF, anterior auditory field; R, rostral; D, dorsal. **(B)** Six tonal receptive fields shown in A. **(C)** Ablation sites in auditory cortex. Images are modified from “The rat brain in stereotaxic coordinates (6th Edition).” Scale bar, 1 mm. **(D)** Schematic drawing of training and testing protocol. **(E)** Freezing duration to sound cues during training session in the first day. **(F)** Comparison of freezing duration between AAF ablated rats (AAF ablated) and normal rats (control). ^∗∗^*p* < 0.01, paired *t*-test; ^∗∗∗^*p* < 0.001, *t*-test.

As precise location of AAF cannot be easily identified anatomically, multiunit extracellular recording was employed to precisely locate AAF in rat auditory cortex (Figures 2A,B). Mirrored distribution of CF can be found in A1 and AAF (Figure 2A). Because the high frequency region of A1 and AAF are colocalized, we focused our investigation in the low frequency region (CF < 10 kHz) in AAF. The typical distance between the low frequency region of A1 and AAF is around ∼2 mm in rat.

To examine the role of AAF in sound cued fear conditioning, low frequency region (CF < 10 kHz) of AAF was ablated bilaterally (Figure 2C). Results of Hematoxylin and eosin (H&E) staining clearly showed ablation of AAF did not damage the A1. Two weeks after AAF ablation, animals were trained and tested using the same protocol shown in Figures 1A, 2D). Without AAF, rats would still freeze when 9 kHz pure tones (dangerous cue) were delivered (Figures 2E,F). The similar learning curve on the 1st training day compared with healthy group suggested that AAF ablation did not significantly harm the learning of fear conditioning (Figure 2E). However, rats without AAF would also freeze when 2 kHz pure tones (safe cue) were delivered which is different from healthy group (AAF ablated: 60.7 ± 7.2%, *n* = 6; control: 26.4 ± 3.2%, *n* = 4, *p* = 0.00006, *t*-test, Figure 2F). In addition, there is no significant (N.S.) difference between the freezing percentage between dangerous cue and safe cue in rats without AAF. These results suggested that: although AAF ablated animals can recognize the incoming threat based on previous training experience, it has lost the ability to categorize which sound cue is really threatening. Considering that animal can make use of both stimulation sequence (e.g., first stimulus, second stimuli) and sound frequency to discriminate different sound cues in training session, but can only make use of sound frequency to categorize in testing session, the normal training curve (Figure 2D) and abnormal testing result (Figure 2E) indicated that AAF ablated animal cannot categorize different sound cues based on their frequencies in fear conditioning task.

### Reversible Inhibition of AAF at Either Training or Testing Session Lead to Similar Results of AAF Ablation

Because AAF ablation is non-reversible, it is unclear if the behavioral abnormality shown in Figure 2 was caused by the loss function of AAF during the training session (memory formation) or testing session (memory retrieval). To better unravel the role of AAF, here we used the chemogenetic tool hM4Di, an engineered Gi protein-coupled receptor activated by the inert ligand clozapine-N-oxide (CNO), to reduce the activity of the neurons in AAF at training or testing session. Adeno associated virus (AAV, pAAV-hSyn-HA-hM4D(Gi)-mCherry) was used to introduce the hM4Di. For control group, another AAV (rAAV-Ef1α-EYFP-wPRE-PA) without functioning receptor was used (see section “Materials and Methods” for details).

**FIGURE 3:**
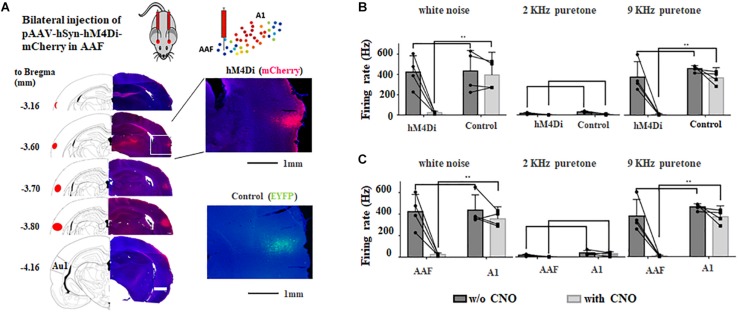
Bilateral chemogenetic inhibition of AAF in adult rats. **(A)** Injection sites of AAVs in adult rats. Images are modified from “The rat brain in stereotaxic coordinates (6th Edition).” Inlets show the fluorescent signals and at injection sites. hM4Di, mCherry; control, EYFP. **(B)** Change of firing rate to different sounds (white noise, 9 and 2 kHz pure tones) in AAF of hM4Di and control group with and without chemogenetic activation (application of CNO). **(C)** Change of firing rate to different sounds (white noise, 9 kHz and 2 kHz pure tones) in AAF and A1 of hM4Di group with and without chemogenetic activation (application of CNO). ^∗∗^*p* < 0.01, *t*-test.

Four weeks after bilateral injection of AAV, both hM4Di and control group showed clear fluorescent signal in AAF but no A1 (Figure 3A), suggesting precise manipulation of AAF is possible. To further validate the effect of chemogenetic inhibition, *in vivo* electrophysiological approach (extracellular recording) was used to compare the neural activities in AAF and A1 before and after hM4Di activation using intraperitoneal injection of CNO (5 mg/Kg). Figure 3B showed that in AAF (CF = 9 kHz), significant decrease of neural firing was observed in hM4Di group to both white noise and 9 kHz pure tones (white noise: from 255.0 ± 95.0 Hz to 14.3 ± 5.9 Hz, 9 kHz: from 225.3 ± 90.9 Hz to 5.0 ± 2.9 Hz). Weak or no response was found to 2 kHz pure tones. In the other hand, no change of firing rate was found in A1 (CF = 9 kHz) with or without CNO injection (Figure 3C). These comparisons suggested that hM4Di inhibition in AAF is both effective and precise.

**FIGURE 4:**
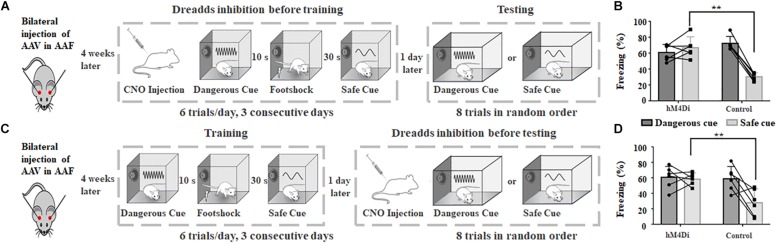
Chemogenetic inhibition of AAF in adult rats impairs animal’s categorization of dangerous and safe cue. **(A)** Schematic drawing of training protocol. Chemogenetic inhibition was activated before training. **(B)** Comparison of freezing duration between hM4Di and control group. CNO was applied before training. **(C)** Schematic drawing of training protocol. Chemogenetic inhibition was activated before testing. **(D)** Comparison of freezing duration between hM4Di and control group. CNO was applied before testing. ^∗∗^*p* < 0.01, *t*-test.

Four weeks after bilateral injection of AAV, both hM4Di and control groups were trained and tested, with CNO injection either before training or testing session (Figures 4A,C). Chemogenetic inhibition at the training session showed similar result to that of AAF ablation (hM4Di: 67.2 ± 12.9%, *n* = 6; control: 30.3 ± 4.9%, *n* = 6, *p* = 0.00047, *t*-test, Figure 4B). Animal can still learn the relationship between sound and incoming threat. However, it would also freeze when the safe cue was delivered. Different from AAF ablation, the cortical circuits in AAF is intact and functioning in the testing session. Without functioning AAF, animal can successfully build the memory about dangerous cue but not the memory of safe cue (hM4Di: 58.5 ± 7.7%, *n* = 6, control: 28.1 ± 17.12%, *n* = 6, *p* = 0.0057, *t*-test, Figure 4D). The memory of safe cue has been categorized or wired incorrectly to the dangerous category. Figure 4D showed the results when chemogenetic inhibition was induced during testing session. Still, there is no major difference compared with the results of AAF ablation. The animal would still freeze to 2 kHz safe cue. Even with correctly formed memory of dangerous and safe sounds, animal without functioning AAF would still fail to categorize sound cues with different frequencies. Taken together, results from Figures 4B,D suggested that AAF might not directly determine the memory formation of sound cue fear conditioning, it might play an important role in categorizing different sound frequencies. Our results showed that when AAF is inactivated (ablated or DREADDS inhibition), animals cannot correctly recognize warning/safe sounds based on sound frequency (Figures 2F, 4B). But during the training session, animals can perform pretty well when fixed training sequence were delivered even if AAF was totally ablated (Supplementary Figure S1). These results suggested that animal may identify warning/safe cues based on sound sequence. But when the sounds are delivered in random order as we did in testing session, animals would not be able to recognize different sound cues based on frequency alone. This supports our hypothesis that AAF is critical for categorization of sound frequency.

## Discussion

### Different Auditory Pathway in Sound Cued Fear Conditioning

Sound cued fear conditioning is one of the most widely used paradigms in the study of learning and memory (Peter et al., 2012; Takemoto and Song, 2019). After several trials of paired delivery of neutral sound (conditioned stimulus, CS) and aversive foot shock (unconditioned stimulus, US), most animals can quickly learn to respond (freeze) while CS was delivered alone (Romanski and LeDoux, 1992). Previous studies have revealed while basolateral amygdaloid nucleus (BLA) is the core nucleus of sound cued fear conditioning, two distinct circuits are involved in this particular task: a cortical pathway and a subcortical pathway (Grosso et al., 2015). For the subcortical pathway, which arises from the medial division of medial geniculate nucleus (MGm), mainly provide animals with fast and ambiguous transmission of auditory information. On the other hand, the cortical pathway, which links the ventral part of medial geniculate nucleus (MGv), primary auditory cortex and amygdala (Boatman and Kim, 2006), may play a role in the discrimination between fear and neutral information (Antunes and Moita, 2010).

In auditory cortex, different subregions might play distinct roles in dissecting properties of sound stimuli. Studies have shown that A1 lesion does not change the establishment and extraction of auditory-related fear memory in animals, while the damage of secondary auditory cortex affects the long-term auditory fear memory of animals (Sacco and Sacchetti, 2010). Meanwhile the role of AAF in sound cued fear conditioning has not been documented. In this study, we found that while animal does not need AAF to establish the link between sound and US (e.g., foot shock) in fear condition task (Figure 2), categorization of different sound frequencies is highly dependent on AAF. Reversible inhibition of AAF using chemogenetic activation at either training or testing session can both lead to strong yet non-selective sound cued freezing behavior. Please be noted that due to the bidirectional interaction between A1 and AAF (Carrasco and Lomber, 2009), change of neuron activities in A1 caused by AAF malfunction may also participated in the abnormal categorization of different frequencies in sound cued fear conditioning.

### Gating Role of AAF Through Selective Inhibition of Fear to Safe Cue

For example, A1 has long been considered as the primary target of MGB and is the most important relay for cortical dependent processing (Budinger et al., 2000). Secondary auditory cortex (A2), on the other hand, have direct projection to subcortical nucleus such as amygdala and has been considered as a center for long-term fear memory (Sacco and Sacchetti, 2010). Different from A1 and A2, previous works including our own results suggested that AAF has abundant connections to ipsilateral and contralateral A1 and contralateral AAF (Budinger et al., 2000; Zhang et al., 2017). Because AAF ablation/inactivation did not block the freezing behavior it is possible that a non-AAF dependent pathway could be the on/off center in sound cued fear conditioning. Thus, the role of AAF is more like a modulatory center for handling the content in sound (e.g., sound frequency) in sound cued fear conditioning. Our results showed that animals are confused and feel danger when a safe sound was heard while AAF is not functioning properly. Interestingly, these animals can behave pretty normal to safe cue during the training sessions (see learning curves in Figure 2 and Supplementary Figure S1). These results raised a possibility that functioning AAF can selectively inhibit fearing behavior to safe cue. And loss of AAF function will results in disinhibition which elicits non-selective freezing behavior to both dangerous and safe cues. While AAF is not the decisive core, it is playing a gating role through selective inhibition of fear to safe cue. While the detailed circuits and cell types are still unclear in this study, the gating effect of AAF is critical for sound categorization in fear conditioning task of adult rat.

## Data Availability Statement

The datasets generated for this study are available on request to the corresponding author.

## Ethics Statement

The animal study was reviewed and approved by the Animal Care and Use Committee of Army Medical University. Written informed consent was obtained from the owners for the participation of their animals in this study.

## Author Contributions

YZ and YX designed and supervised the study. ZS, SY, SQ, CZ, and ZW performed the experiments. ZS and SY performed the data analysis. ZS, YD, and YZ prepared the figures and wrote the manuscript. YDZ, HW, PC, QD, and TL helped for revising the manuscript. CG, MZ, and YJZ were undergraduate students from Chongqing University of Science and Technology. They helped to build the training and testing chambers used in this study.

## Conflict of Interest

The authors declare that the research was conducted in the absence of any commercial or financial relationships that could be construed as a potential conflict of interest.
