# Supplementary figures and images for: Anterior Auditory Field Is Needed for Sound Categorization in Fear Conditioning Task of Adult Rat
Source: Front Neurosci. 2019 Dec 20;13:1374. doi: 10.3389/fnins.2019.01374 (PMC6933002; doi:10.3389/fnins.2019.01374)

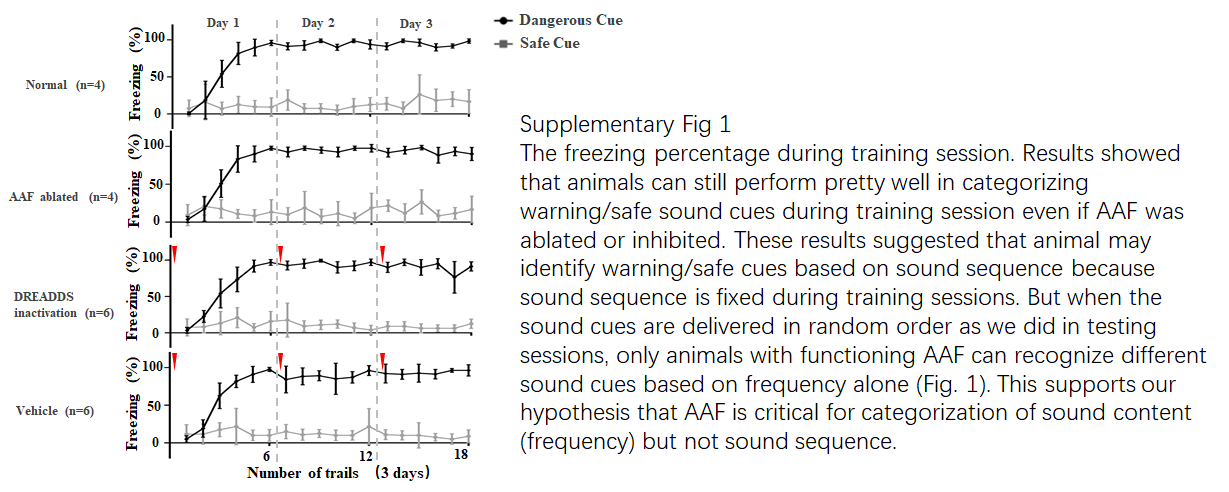

Supplement: Supplementary file 1 [file Image_1.TIF]
